# Changes in Centrality Frequency of the Default Mode Network in Individuals With Subjective Cognitive Decline

**DOI:** 10.3389/fnagi.2019.00118

**Published:** 2019-06-20

**Authors:** Yunyan Xie, Tiantian Liu, Jing Ai, Duanduan Chen, Yiran Zhuo, Guanglei Zhao, Shuai He, Jinglong Wu, Ying Han, Tianyi Yan

**Affiliations:** ^1^Department of Neurology, Xuanwu Hospital, Capital Medical University, Beijing, China; ^2^School of Life Science, Beijing Institute of Technology, Beijing, China; ^3^College of Electronic and Information Engineering, Tongji University, Shanghai, China; ^4^Beijing Haidian Foreign Language Shiyan School, Beijing, China; ^5^School of Mechatronical Engineering, Intelligent Robotics Institute, Beijing Institute of Technology, Beijing, China; ^6^Center of Alzheimer's Disease, Beijing Institute for Brain Disorders, Beijing, China; ^7^Beijing Institute of Geriatrics, Beijing, China; ^8^National Clinical Research Center for Geriatric Disorders, Beijing, China

**Keywords:** subjective cognitive decline, centrality frequency, resting-state functional magnetic resonance imaging, hub probability, default mode network

## Abstract

Despite subjective cognitive decline (SCD), a preclinical stage of Alzheimer's disease (AD), being widely studied in recent years, studies on centrality frequency in individuals with SCD are lacking. This study aimed to investigate the differences in centrality frequency between individuals with SCD and normal controls (NCs). Forty individuals with SCD and 53 well-matched NCs underwent a resting-state functional magnetic resonance imaging scan. We assessed individual dynamic functional connectivity using sliding window correlations. In each time window, brain regions with a high degree centrality were defined as hubs. Across the entire time window, the proportion of time that the hub appeared was characterized as centrality frequency. The centrality frequency correlated with cognitive performance differently in individuals with SCD and NCs. Our results revealed that in individuals with SCD, compared with NCs, correlations between centrality frequency of the anterior cortical regions and cognitive performance decreased (79.2% for NCs and 43.5% for individuals with SCD). In contrast, correlations between centrality frequency of the posterior cortical regions and cognitive performance increased in SCD individuals compared with NCs (20.8% for NCs and 56.5% for individuals with SCD). Moreover, the changes mainly focused on the anterior (93.3% for NCs and 45.5% for individuals with SCD) and posterior (6.7% for NCs and 54.5% for individuals with SCD) regions associated with the default mode network (DMN). In addition, we used absolute thresholds (correlation efficient *r* = 0.2, 0.25) and proportional thresholds (sparsity = 0.2, 0.25) to verify the results. Dynamic results are relative stable at absolute thresholds while static results are relative stable at proportional thresholds. Converging findings provide a new framework for the detection of the changes occurring in individuals with SCD via centrality frequency of the DMN.

## Introduction

Alzheimer's disease (AD) is one of the classical chronic neurodegenerative diseases and considered of the common cause of dementia. With the progression of the disease, patients gradually lose independence and withdraw from family and society. According to the Alzheimer's Association Report, the patients' medical care cost is up to 232 billion dollars in America in 2017 (Alzheimer's, 2018). Moreover, there are no effective treatments that can stop or slow AD progression by much (Winblad et al., 2016), so developed countries have spent a great deal on AD patients' medical care. Subjective cognitive decline (SCD) appears at a very early stage of AD and has the potential to be an effective symptomatic indicator of preclinical AD (Lopez-Sanz et al., 2017). In clinical trials, SCD generally refers to subjectively experienced cognitive deterioration (Tales et al., 2015) and primarily related to the increasing risk of developing AD (Jessen et al., 2014). Convergent evidence from clinical studies shows that tau, Aβ protein levels and gray matter atrophy are the current validated biomarkers for the early identification of AD (Lopez-Sanz et al., 2018). In addition, researchers found a disrupted pattern in the peripheral brain regions of SCD subjects based on the structural diffusion tensor imaging data (Yan et al., 2018). However, the International Working Group has reported that structural and metabolic changes emerge later than functional changes (Dubois et al., 2016), which can be assessed by electrophysiological techniques or magnetic resonance imaging (MRI) scans. Previous studies have shown abnormal increased brain activity during memory tasks in MCI (Puregger et al., 2003) and SCD (Maestu et al., 2011) subjects. Even in spontaneous brain activity, SCD individuals have presented with significant alpha power alterations (Lopez-Sanz et al., 2016). The above studies mainly adopted the method of magnetoencephalogram (MEG) or electroencephalogram (EEG) because of its high temporal resolution.

MRI scans have higher spatial resolution, which is better for simultaneously knowing about the brain structure and function. In recent years, resting state functional magnetic resonance imaging (fMRI) has attracted more and more attention on the application of studying the mechanisms of neurological disorders (Lau et al., 2016). Resting state fMRI, which can reflect intrinsic brain activity, is based on a blood oxygenation level-dependent (BOLD) signal to extract biomarkers. Recently, resting state fMRI has been effectively used for the preclinical identification of AD (Wee et al., 2013). One common method to process resting state fMRI data is functional connectivity (FC), which sheds light on the exchange of information across functionally specialized brain regions. This method usually calculates the pairwise Pearson's correlation coefficient or the sparse representation (Jie et al., 2014) between every pair of *N* time series from averaged brain regions to obtain a whole brain FC (Zhang et al., 2017a). Compared with the sparse representation measures, Pearson's correlation is more easily calculated and more widely used (Chen et al., 2016). Based on this method, researchers found that in MCI subjects, not only functional connectivity between the left thalamus and a set of regions was decreased (Wang et al., 2012) but also functional connectivity in cortical midline structures was decreased (Ries et al., 2010). Besides, the whole-brain voxel-wise degree map measured by static functional connectivity also showed the reduced degree in the right middle occipital gyrus in the progression from MCI to AD (Deng et al., 2016). In addition, Vega et al. (2016) found that elderly SCD women who reported more severe cognitive decline showed weaker negative functional connectivity within the frontal cortex and stronger positive connectivity within the right middle temporal gyrus. Thus, abnormal FC across specific brain regions has been associated with cognitive decline, which supports the “disconnection” hypothesis in SCD.

The functional connectivity studies above are almost based on a static functional network, which represents an average and is stable. However, some findings have suggested that the brain functional connectivity is non-stationary and not in a state of equilibrium, and the discrete FC states switch rapidly (Allen et al., 2014; Hansen et al., 2015; Vidaurre et al., 2017). Therefore, compared with traditional average functional connectivity, dynamic functional connectivity provides a new perspective for data analysis. Resting state brain networks (RSNs) constructed by MEG and fMRI showed significant similarity (Brookes et al., 2011), but different stationarity (de Pasquale et al., 2010). MEG RSNs showed more non-stationary maybe because of the high time resolution. Thus, researchers studied the temporal dynamics of hub regions at the slow and fast timescale measured by MEG (de Pasquale et al., 2012, 2016, 2018; Betti et al., 2018). Dynamic analysis is often used under the assumption that the relationships between areas are of greater interest than the relative signal amplitudes (Keilholz et al., 2017). A dynamic functional network can reveal the rapid fluctuation and time-varying characteristics of brain function, which cannot be revealed in the static functional connectivity analysis. The dynamic FC analysis has great potential in the field of neurological disorders, providing biomarkers in major disorders and diseases (Kaiser et al., 2016), including schizophrenia (Damaraju et al., 2014), Parkinson's disease (Rowe et al., 2010), and Alzheimer's disease (Jones et al., 2012). In a study of particular note, researchers showed that compared with features from static functional connectivity, using features selected from a dynamic network can achieve better performance in discriminating AD patients from normal controls (de Vos et al., 2018). Thus, dynamic connectivity has the potential to find optimal disease markers. However, to our knowledge, few studies have focused on dynamic functional connectivity in SCD (Pijnenburg et al., 2008; Jiang et al., 2018).

In this study, we constructed dynamic resting state FC with fMRI of normal control participants and SCD participants. A previous study showed the disruption of average FC network with MEG in SCD participants and further demonstrated the different synchronization patterns in anterior default mode network (DMN) and posterior DMN compared to normal control participants (Lopez-Sanz et al., 2017). The above results were based on static FC, while no dynamic FC reported similar results according to our knowledge. Additionally, a previous study from our group showed the rich club disturbances in SCD participants, which indicated the abnormality of highly connected hubs (Yan et al., 2018). Hence, we hypothesized that the disruption of FC in SCD participants not only in static FC, but also in dynamic FC. And the disruption of highly connected hubs revealed by dynamic FC would take place in the SCD stage. In addition, in order to compare with previous studies, we classified the hubs into anterior and posterior network, DMN and non-DMN.

## Methods

### Participants

The dataset in this study has been reported by previous study (Yang et al., 2018). This study was approved by the Medical Research Ethics Committee and Institutional Review Board of Xuanwu Hospital (ClinicalTrials.gov identifier: NCT02353884 and NCT02225964). A total of 93 Chinese participants, including 53 normal control (NC) participants and 40 SCD participants with memory concerns were voluntarily recruited for this study. NC participants were recruited by advertisements and SCD participants were recruited from the memory clinic of the Medical Neurology Department of Xuan Wu Hospital Capital Medical University in Beijing, China. All participants agreed to and signed the informed consent in accordance with the Declaration of Helsinki. The diagnosis of SCD was consistent with SCD Initiative (Jessen et al., 2014). Experienced neurologists evaluated all the participants by professional cognitive scales, including the Chinese version of the Mini-Mental State Examination (MMSE), the Beijing version of Montreal Cognitive Assessment (MoCA), the auditory verbal learning test (AVLT), the Clinical Dementia Rating (CDR), the clock drawing test (CDT), Activities of Daily Living (ADL) Scale, Hachinski Ischemic Score (HIS), and Center for Epidemiologic Studies depression scale (CES-DS). All the participants had the MMSE and MoCA scores with the normal range as reported in the previous study (Yang et al., 2018). NC participants had no memory concerns, while SCD participants had self-report continuous memory decline within the last 5 years and confirmed by an informant. And demographic details and participants' scores on neuropsychological tests are summarized in Table 1.

**Table 1:** Participant demographics.

**Group**	**NC (*n* = 53)**	**SCD (*n* = 40)**	***P*-value**
Age (years)	63.50 ± 8.25	64.90 ± 8.31	0.421
Gender (M/F)	21/32	16/24	0.900
Education	10.98 ± 5.10	11.65 ± 4.53	0.513
AVLT-I	9.23 ± 1.89	8.32 ± 1.92	0.027
AVLT-D	10.15 ± 2.87	8.95 ± 2.66	0.043
AVLT-R	12.00 ± 2.61	11.18 ± 2.75	0.144
MMSE	28.19 ± 2.17	28.05 ± 1.93	0.750
MoCA	26.22 ± 3.16	25.51 ± 1.73	0.288
CDT	2.64 ± 0.65	2.57 ± 0.69	0.604
CDR	0.00 ± 0.00	0.01 ± 0.08	0.324
CES-DS	2.18 ± 4.59	4.52 ± 5.59	0.073
HIS	0.06 ± 0.32	0.62 ± 1.72	0.058
ADL	20.02 ± 0.14	20.28 ± 1.26	0.228

*The P-value for gender distribution in the two groups was obtained by the chi-squared test. The P-values for differences in age, years of education and scale scores between the two groups were obtained by the two-sample t-test. Values are expressed as the mean ± SD*.

### MRI Acquisition

All participants were imaged with a 3.0 Tesla MR imager (Siemens Magnetom Trio Tim MRI system, Germany) using a standard head coil. Resting-state blood oxygenation level dependent (BOLD) signals were collected using an echo-planar imaging (EPI) sequence with the following parameters: 28 axial slices; repetition time (TR) = 2,000 ms; echo time (TE) = 40 ms; flip angle (FA) = 90°; slice thickness = 4.0 mm; gap = 0.8 mm; matrix = 64 × 64; and field of view (FOV) = 256 × 256 mm. All participants were asked to keep their eyes closed and mind relaxed with as little motion as possible during the scan, which lasted for 8 min. In addition to rs-fMRI scans, T1-weighted images were acquired for anatomical reference. T1-weighted MR images were obtained by a 3D magnetization-prepared rapid gradient echo (MPRAGE) with the following parameters: slices = 176, thickness = 1.0 mm, TR = 1,900 ms, TE = 2 ms, inversion time (TI) = 900 ms, FA = 9°, FOV = 224 × 256 mm, and matrix = 448 × 512.

### Data Preprocessing

Based on the MATLAB software platform, the rs-fMRI data were preprocessed via GRETNA (Wang et al., 2015), a graph theoretical network analysis toolbox for imaging connectomics that included the following preprocessing steps. First, we applied the removal of the first 10 time points to improve the signal-to-noise ratio. Second, the slice-time and head motions were corrected for the difference between scan layers. Third, the functional data were normalized into Montreal Neurological Institute (MNI) space by structural images (T1 images) for each subject. Forth, spatial smoothing with a 4 mm full-width half-maximum Gaussian kernel, removal of the linear trend in the signal, band-pass filtering (0.01–0.1 Hz) and global signal regression were applied to data in succession. Lastly, the functional data were transformed to the Automated Anatomical Labeling (AAL) atlas by the LDDMM transformation (Du et al., 2011; Tan and Qiu, 2016).

### Mapping Hubs in Static and Dynamic Functional Connectivity

In static functional connectivity, we divided the brain (not including cerebellum regions) into 90 regions based on the AAL atlas and used the average signal for each region to obtain a functional connectivity matrix. Functional connectivity between all pairs of the 90 regions was represented by linear Pearson correlation coefficients between all pairs of the time series. *R*_*ij*_ was the Pearson correlation coefficient between the ith brain region and the jth brain region:

$$R_{ij}=\frac{\sum \left[\left(x{\left[t\right]}_{i}-\bar{x_{i}}\right)\left(x{\left[t\right]}_{j}-\bar{x_{j}}\right)\right]}{\sqrt{\sum \left[{\left(x{\left[t\right]}_{i}-\bar{x_{i}}\right)}^{2}{\left(x{\left[t\right]}_{j}-\bar{x_{j}}\right)}^{2}\right]}}\;i,j\;=1\ldots 90$$

This will generate a 90 × 90 correlation matrix. To identify hubs, we counted the number of strongly functional connectivity to each ROI (above a threshold of *r* > 0.25). This metric is sometimes referred to as degree centrality or degree in graph theory (Carboni, 2015). This measure of connectivity (degree, D) for each ROI (i) with all other regions (j) is determined as follows:

$$\begin{array}{ll} D_{i}=\sum d_{ij}\;j & =1\ldots 90,\;i\neq j \end{array}$$

Then for the comparison between groups, the degree of each ROI was normalized to *Z*-scores in every correlation matrix. The *Z*-score transformation was as follows:

$$\begin{array}{l} Z_{i}=\frac{D_{i}-\bar{D}}{\sigma_{D}}i=1\ldots 90, \end{array}$$

where $\bar{\text{D}}$ refers to the mean degree across all the ROIs and σ_*D*_ refers to the standard deviation of all the ROIs in every correlation matrix. According to a previous study, hubs were defined based on the degree and the degree was one standard deviation higher than the mean value (Buckner et al., 2009).

In dynamic functional connectivity, 215 brain networks (215 time windows) were constructed for each subject. Combined with the sliding time-window approach, a 90 × 90 brain network was constructed in each time window with the width of 25 TR (50 s) slid in steps of 1 TR (2 s). As mentioned above, the hubs were defined in each time window according to the same algorithm to static functional connectivity. The *Z*-score transformation of degree was performed in each time window. Furthermore, we calculated the percentage of each ROI being hubs during the whole time windows. The percentage was defined as hub probability and suggest the centrality frequency of specific ROI (Zhang et al., 2017b).

### Statistical Analysis

To compare the difference of dynamic functional connectivity between NC and SCD group, we first ran independent *t*-test for hub probability of each ROI. Furthermore, to reveal the relationship between functional brain dynamics and cognitive performance, the multiple linear regression model was employed in NC group, SCD group and all participants. The hub probability of 90 AAL regions was entered as the main factor, and the scores of each neuropsychological tests was entered as the dependent variable, while controlling for age, gender and education level. We selected the brain regions (*p* < 0.05) whose centrality frequency played important roles in cognitive performance for further analyses.

Similar analyses were performed on static functional connectivity. The degree centrality was entered as the main factor for the multiple linear regression model and the brain regions (*p* < 0.05) were selected for the further analyses.

### Validation of the Degree Centrality

The calculation of degree centrality was fundamental to the present analysis. Thus, based on the previous definition of strongly functional connectivity (at a threshold of correlation efficient *r* = 0.25), we ran the similar analysis at the other threshold of correlation efficient *r* = 0.2 to compare the results. In addition, previous studies showed different strategies for the threshold selection to obtain a binary graph, such as a fixed density (Zalesky et al., 2014), multiple densities (Chiang et al., 2016), and individual statistical threshold (de Pasquale et al., 2013, 2017). A previous study reported that the absolute threshold (based on correlation efficient) and proportional threshold (based on sparsity) were popular and powerful to obtain the binary graphs (Garrison et al., 2015). Therefore, we performed Z-scored transformation on the Pearson correlation matrix and ran the similar analysis on the matrix at the proportional threshold (sparsity = 0.2, 0.25) to compare the results (Zhang et al., 2017b).

## Results

### Difference of Hub Probability Between NC and SCD Group

**Figure 1:**
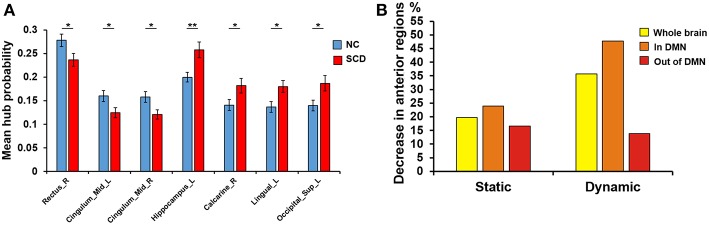
Difference between groups at the absolute threshold (correlation efficient *r* = 0.25). **(A)** The mean hub probability of seven brain regions (right gyrus rectus, left midcingulate area, right midcingulate area, left hippocampus, right calcarine sulcus, left lingual gyrus and left superior occipital) showed significant difference between NC (blue) and SCD (red) group. Statistical significance: ^*^p < 0.05; ^**^p < 0.01. **(B)** The proportion of anterior regions in NC group minus the proportion in SCD group from the three levels: whole brain (yellow), in DMN (orange) and out of DMN (red). Both the static and dynamic functional connectivity results were shown.

We performed independent *t*-test on mean hub probability of each ROI (90 ROIs) between NC and SCD group. As shown in Figure 1A, right gyrus rectus, left midcingulate area, right midcingulate area, left hippocampus, right calcarine sulcus, left lingual gyrus and left superior occipital showed significant difference (*p* < 0.05) between NC and SCD group. The *p*-value is 0.036, 0.035, 0.018, 0.003, 0.029, 0.015, 0.017, respectively.

### Relationship Between Degree Centrality and Neuropsychological Tests

For the static functional connectivity, we performed linear regression analyses between scores on different neuropsychological tests and degree centrality of 90 brain regions, finding 17 brain regions in the NC group, 23 brain regions in the SCD group and 22 brain regions in all participants. The degree centrality of above brain regions was significantly correlated with the scores (*p* < 0.05, see Supplementary Table 1 and Figure 1B). We considered that these brain regions contributed more prominently to cognitive performance.

**Figure 2:**
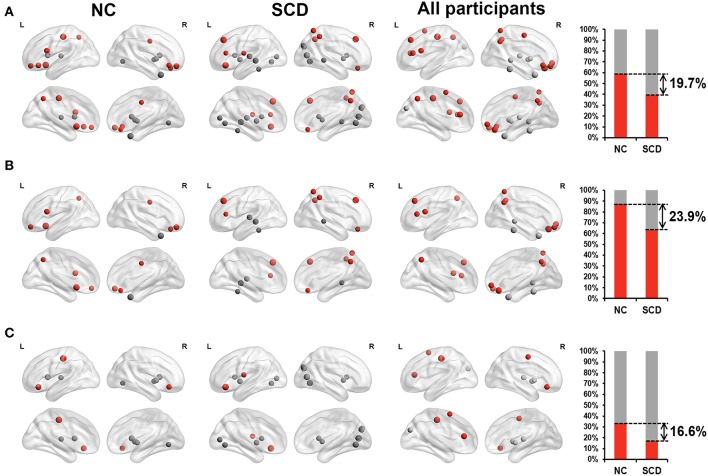
Relationship between *Z*-scored degree centrality measured by static functional connectivity of brain regions and neuropsychological tests in NC group (the first column), SCD group (the second column) and all participants (the third column). The forth column showed the proportion of significant correlated regions (*p* < 0.05) located in anterior (red) cortical and other (gray) cortical regions in the two groups. The nodes covered by red are located in anterior cortical regions. By contrast, nodes covered by gray are located in posterior cortical regions or subcortical regions. The size of each node represents the absolute value of standardized ß. Nodes are located according to their centroid stereotaxic coordinates. **(A)** All the significant correlated regions (*p* < 0.05). **(B)** The regions in the default mode network. **(C)** The regions out of the default mode network.

Furthermore, we classified these brain regions into the anterior region, posterior region and subcortical region. 58.8% of the brain regions we found in the NC group were located in anterior cortical regions, including the bilateral superior frontal gyrus, orbital part, left opercular part of inferior frontal gyrus, bilateral orbital part of inferior frontal gyrus, left olfactory cortex, right gyrus rectus, right middle cingulate, left postcentral gyrus and left precuneus. However, only 39.1% of the brain regions we found in the SCD group were located in anterior cortical regions, including the bilateral superior frontal gyrus, dorsolateral, left area triangularis, left orbital part of inferior frontal gyrus, left rolandic operculum, right gyrus rectus, right superior parietal lobule, right inferior parietal lobule, and right precuneus (see Figure 2). These results suggested that compared with NCs, degree centrality in anterior cortical regions decreased its contribution to cognitive performance in SCD participants.

### Relationship Between Hub Probability and Neuropsychological Tests

For the dynamic functional connectivity, we performed linear regression analyses between scores on different neuropsychological tests and hub probabilities of 90 brain regions, finding 24 brain regions in the NC group, 23 brain regions in the SCD group and 24 brain regions in all participants. The hub probability of above brain regions was significantly correlated with the scores (*p* < 0.05, see Supplementary Table 2 and Figure 1B). We considered that these brain regions contributed more prominently to cognitive performance.

**Figure 3:**
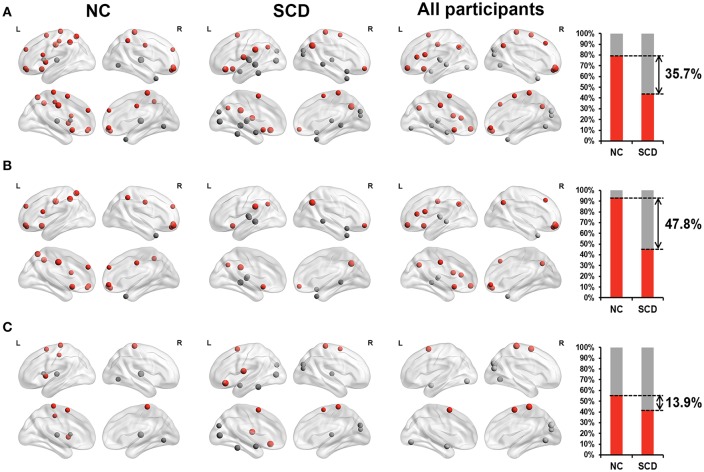
Relationship between hub probability measured by dynamic functional connectivity of brain regions and neuropsychological tests in NC group (the first column), SCD group (the second column) and all participants (the third column). The forth column showed the proportion of significant correlated regions (*p* < 0.05) located in anterior (red) cortical and other (gray) cortical regions in the two groups. The nodes covered by red are located in anterior cortical regions. By contrast, nodes covered by gray are located in posterior cortical regions or subcortical regions. The size of each node represents the absolute value of standardized ß. Nodes are located according to their centroid stereotaxic coordinates. **(A)** All the significant correlated regions (*p* < 0.05). **(B)** The regions in the default mode network. **(C)** The regions out of the default mode network.

Furthermore, we classified these brain regions into the anterior region, posterior region and subcortical region. 79.2% of the brain regions we found in the NC group were located in anterior cortical regions, including the bilateral superior frontal gyrus (orbital part),bilateral middle frontal gyrus, orbital part, left opercular part of inferior frontal gyrus, left supplementary motor area, left olfactory cortex, bilateral superior frontal gyrus, medial part, right superior frontal gyrus, medial orbital part, left insula, bilateral middle cingulate, left postcentral gyrus, left superior parietal lobule, bilateral inferior parietal lobule, and bilateral paracentral lobule. However, only 43.5% of the brain regions we found in the SCD group were located in anterior cortical regions, including the right middle frontal gyrus, orbital part, left orbital part of inferior frontal gyrus, left rolandic operculum, bilateral supplementary motor area, left olfactory cortex, left supramarginal gyrus, bilateral angular gyrus and right paracentral lobule (see Figure 3A). These results suggested that compared with NCs, centrality frequency in anterior cortical regions decreased its contribution to cognitive performance in SCD participants.

Similar changes were also found in the default mode network (DMN). We classified the brain regions into the DMN (Andrews-Hanna et al., 2014) and other regions. Both the NC group and SCD group had approximately 50% of the identified brain regions belong to the DMN. Within these regions, 93.3% of them belonged to anterior cortical regions in the NC group, including the bilateral superior frontal gyrus (orbital part), bilateral middle frontal gyrus, orbital part, left opercular part of inferior frontal gyrus, left olfactory cortex, bilateral superior frontal gyrus, medial part, right superior frontal gyrus, medial orbital part, bilateral middle cingulate, left superior parietal lobule and bilateral inferior parietal lobule. Only 45.5% of them belonged to anterior cortical regions in the SCD group, including the right middle frontal gyrus, orbital part, left olfactory cortex, left supramarginal gyrus and bilateral angular gyrus (see Figure 3B). The distribution of the brain regions out of the DMN did not show such large differences between the NC group and the SCD group (see Figure 3C). According to the above results, we hypothesized that centrality frequency in anterior cortical regions, especially in the DMN, decrease their contribution to cognitive performance in the transition from NC to SCD.

Since the SCD group showed decreased contribution to cognitive performance in the anterior cortical regions, we showed the difference of anterior cortical regions in the whole brain, in DMN and out of DMN (the proportion of anterior cortical regions in NC group minus the proportion in SCD group, see Figure 3B). The regions in DMN showed the largest proportion both in static and dynamic functional connectivity.

### Validation

#### Validation of the Degree Centrality

**Figure 4:**
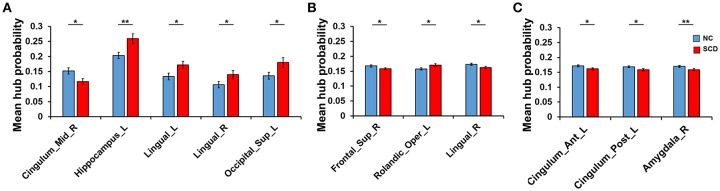
Difference between groups at the different thresholds. Statistical significance: ^*^p < 0.05; ^**^p < 0.01. **(A)** The absolute threshold (correlation efficient r=0.2). The mean hub probability of five regions (right midcingulate area, left hippocampus, left and right lingual gyrus and left superior occipital) showed significant difference between NC (blue) and SCD (red) group. **(B)** The proportional threshold (sparsity = 0.2). Right superior frontal gyrus, left rolandic operculum, and right lingual gyrus showed significant difference. **(C)** The proportional threshold (sparsity = 0.25). Left anterior cingulate gyrus, left posterior cingulate gyrus, and right amygdala showed significant difference.

Differences of hub probability between NC and SCD group in different conditions were shown in Figure 4. Five brain regions showed significant differences (*p* < 0.05) at a threshold of correlation efficient *r* = 0.2. And the five regions were included in the regions revealed at a threshold of correlation efficient *r* = 0.25. As for the binary network with sparsity of 0.2 and 0.25, only three brain regions showed significant differences (*p* < 0.05) between the two groups, respectively. The brain regions were right superior frontal gyrus, left rolandic operculum, and right lingual gyrus for the sparsity of 0.2 and left anterior cingulate gyrus, left posterior cingulate gyrus, right amygdala for the sparsity of 0.25. Specifically, the regions revealed by sparsity algorithm with the two thresholds were totally different.

**Figure 5:**
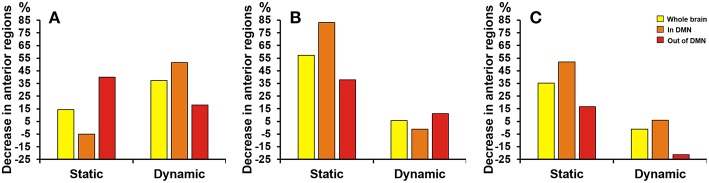
Proportion of SCD group's decrease in anterior cortical regions relative to NC group. The proportion of anterior regions revealed by static and dynamic functional connectivity in NC group minus the proportion in SCD group from the three levels: whole brain (yellow), in DMN (orange) and out of DMN (red). **(A)** The absolute threshold (correlation efficient *r* = 0.2). **(B)** The proportional threshold (sparsity = 0.2). **(C)** The proportional threshold (sparsity = 0.25).

As Figure 5 shown, differences at a threshold of correlation efficient *r* = 0.2 were similar to the threshold of correlation efficient *r* = 0.25 in the dynamic functional connectivity. However, the static functional connectivity showed different trends. For the static functional connectivity, the regions out of DMN decreased more than regions in DMN with the threshold of correlation efficient *r* = 0.2 and the results were opposite with the threshold of correlation efficient *r* = 0.25. For the sparsity algorithm, the static functional connectivity showed similar trends with sparsity of 0.2 and 0.25, but the dynamic functional connectivity showed different trends. More details were shown in Supplementary Material about the multiple regression model results of static and dynamic functional connectivity with the threshold of correlation efficient *r* = 0.2, sparsity of 0.2 and 0.25 (Supplementary Tables 3–5).

## Discussion

The aim of the present study was to determine the differences in the centrality frequency of the default mode network (DMN) in individuals with subjective cognitive decline (SCD). The results were consistent with the hypothesis that SCD individuals showed obvious abnormalities in centrality frequency in an anterior-posterior distribution and the abnormality was related to cognitive performance. In particular, our results revealed that, compared with the NC group, the percentage of brain regions in the SCD group whose hub probabilities were significantly correlated with the scores on the neuropsychological tests obviously decreased in anterior cortical regions and increased in posterior cortical regions. Moreover, this phenomenon occurred mainly in the default mode network (DMN). The results were consistent in static and dynamic functional connectivity. Furthermore, we used the threshold of correlation efficient *r* = 0.2, sparsity of 0.2 and 0.25 to verify the results. Results showed that sparsity algorithm was stable to the static functional connectivity, while the threshold algorithm was stable to dynamic functional connectivity. Based on our results, we might say that regarding centrality frequency, the DMN is more susceptible to damage compared to other regions in SCD individuals.

### Difference of Hub Probability Between NC and SCD Group

Seven brain regions showed significant different hub probability between NC and SCD group. Among them, right gyrus rectus, left midcingulate area and right midcingulate area showed smaller hub probability in SCD group. However, left hippocampus, right calcarine sulcus, left lingual gyrus and left superior occipital showed larger hub probability in SCD group compared to NC group. Similar to previous studies, researchers have showed that gyrus rectus and hippocampus were important MRI biomarkers for the early diagnosis of Alzheimer's disease by machine learning (Salvatore et al., 2015). Besides, researchers revealed that cingulate and calcarine sulcus showed the opposite correlations between sulcal variability and cognition in Alzheimer's brain (Mega et al., 1998). A resting-state functional connectivity study showed that connectivity in the lingual gyrus and occipital was related to subjective memory complaints severity (Kawagoe et al., 2019). Converging results indicated that different brain regions showed different centrality frequency patterns in the SCD group, which might help with the early detection of cognitive impairment.

### Anterior Brain Region Abnormalities Indicated by Centrality Frequency

Anterior regions, including the dorsolateral prefrontal cortex, medial prefrontal cortex, anterior cingulate cortex, posterior cingulate cortex, precuneus, sensorimotor cortex and lateral parietal cortex, play an important role in episodic memory, mental activity, movement, and social behavior (Devinsky et al., 1995; Davidson et al., 2008). A recent study showed that in SCD individuals, anterior and posterior regions behaved differently in the pattern of alterations. In particular, hyper synchronization over anterior regions and hypo synchronization over posterior regions (Lopez-Sanz et al., 2017). Our study suggested that centrality frequency in anterior regions can weaken its contribution to cognitive performance in SCD individuals. The present results were similar to some previous studies that have found abnormalities in the above regions in Alzheimer's disease and its early stage, such as astrocyte metabolic reduction in PCC (Minoshima et al., 1997) and excitability enhanced in SMC (Ferreri et al., 2016). Both the previous studies and our study showed that at the global spatial pattern level, the abnormalities of anterior brain regions have been highlighted in SCD individuals.

### DMN Abnormalities Indicated by Centrality Frequency

The DMN, consisting of discrete, bilateral and symmetrical cortical areas, plays a central role in the brain's intrinsic activity. Functions of the DMN include scene construction, associative prediction, episodic memory processing, self-processing, mentalizing and conceptual processing (Andrews-Hanna et al., 2014). Our results implied that in individuals with SCD, the centrality frequency of DMN changed more than in other regions. Many existing studies have also discovered abnormalities in the DMN in Alzheimer's disease and its incipient stage. Dillen et al. (2017) revealed that the hippocampus functionally decoupled from posterior DMN nodes in SCD and prodromal AD patients. Besides, Jones et al. (2011) found that AD patients displayed an accelerated pattern of age-associated changes in the DMN, such as declining connectivity in the posterior DMN. Su et al. (2017) suggested that the breakdown of DMN connectivity may occur in the very early stage of Alzheimer's disease. Taken together, these studies emphasized the abnormal functional connectivity pattern of the DMN in the progression of AD.

Our study revealed abnormalities in SCD via dynamic functional connectivity. Previous studies focused more on brain network feature extraction and then found abnormalities in AD patients with permutation entropy (Wang et al., 2017), minimum spanning tree (Wang et al., 2018), rich club structures (Yan et al., 2018), and so on. In recent years, dynamic FC analysis has been used to provide functional biomarkers for Alzheimer's disease, even its early stage. Jones et al. (2012) adopted a functional connectivity graph based on the sliding time window method to study the dynamic abnormal spontaneous activity of the brain in patients with AD and found that links in the posterior DMN were significantly reduced in AD patients. What's more, Quevenco et al. (2017) indicated that alteration of anterior-posterior brain dynamics were related to memory abnormality in the preclinical stage of AD. In a recent study, researchers (de Vos et al., 2018) also found that compared with static functional connectivity features, using dynamic functional connectivity features to classify AD patients and normal controls resulted in a better classification level. Based on dynamic brain networks, these findings provided a new perspective for the rs-fMRI data analysis of SCD individuals.

## Conclusion

Above all, our study provided a new point of view to detect the changes occurring in SCD with resting-state fMRI. In SCD individuals, centrality frequency in the anterior cortical regions weakened its contribution to cognitive performance, especially in the default mode network. Therefore, it is of great significance to explore the individual development and phylogeny of the default mode network in a resting state. The other striking aspect of the data analysis was that we used threshold and sparsity algorithm with different parameters (0.2 and 0.25) to verify our results. A previous MEG study used threshold and sparsity algorithms to obtain the binary network (de Pasquale et al., 2016). They indicated that threshold algorithm could avoid the influence of large number of small weights. In the present study, the static functional connectivity was fluctuant with different thresholds (0.2 and 0.25). Thus, we speculated that the static functional connectivity had large number of small weights. In this study, some limitations must be emphasized. First of all, the selection process of participants was quite subjective and need to be improved. Second, the data processing parameters could have been chosen more carefully, such as the length of the window. Third, the transformation of SCD to MCI or AD is not a certain outcome in the current study. Therefore, in the future, we need to perform more follow-up analyses to confirm the transition of SCD individuals and try to predict the progression of the disease with centrality frequency of the DMN.

## Ethics Statement

All participants agreed to and signed the informed consent in accordance with the Declaration of Helsinki. This study was approved by the Medical Research Ethics Committee and Institutional Review Board of Xuanwu Hospital.

## Author Contributions

In this study, YX recruited participants and wrote the manuscript. TL analyzed the data and wrote the manuscript. JA and SH did the preprocessing of data. DC revised the manuscript. YZ and GZ revised the manuscript and helped with the data analysis. JW corrected the grammar. YH collected the data. TY provided data analysis ideas.

### Conflict of Interest Statement

The authors declare that the research was conducted in the absence of any commercial or financial relationships that could be construed as a potential conflict of interest.
