# Supplementary material for: Changes in Centrality Frequency of the Default Mode Network in Individuals With Subjective Cognitive Decline
Source: Front Aging Neurosci. 2019 Jun 20;11:118. doi: 10.3389/fnagi.2019.00118 (PMC6595963; doi:10.3389/fnagi.2019.00118)
Supplement: Supplementary file 1 [file Table_2.DOCX]

Supplementary Material

Changes in functional dynamics of the default mode network in individuals with subjective cognitive decline

**Yunyan Xie^1✝^, Tiantian Liu^2✝^, Jing Ai^2^, Duanduan Chen^2^, Shuai He^3^, Jinglong Wu^4^, Ying Han^1*^, Tianyi Yan^2*^**

^1^Department of Neurology, Xuanwu Hospital, Capital Medical University, Beijing, China

^2^School of Life Science, Beijing Institute of Technology, Beijing, China

^3^Beijing Haidian Foreign Language Shiyan School, Beijing, China

^4^School of Mechatronical Engineering, Intelligent Robotics Institute, Beijing Institute of Technology, Beijing, China

*** Correspondence:**

Ying Han
hanying@xwh.ccmu.edu.cn

Tianyi Yan
yantianyi@bit.edu.cn

^✝^These authors have contributed equally to this work.

# Supplementary Tables

## Supplementary Table 1: Linear regression model results of static functional connectivity with the threshold of r > 0.25.

| Brain regions | NC | | | SCD | | | All participants | | |
| --- | --- | --- | --- | --- | --- | --- | --- | --- | --- |
|  | Neuropsychology test | Standardized-β | p-values | Neuropsychology test | Standardized-β | p-values | Neuropsychology test | Standardized-β | p-values |
| Precentral_R |  |  |  |  |  |  | AVLT-D | -0.219 | 0.035* |
| Frontal_Sup_L |  |  |  | AVLT-D | -0.475 | 0.002** | AVLT-I | -0.237 | 0.023* |
|  |  |  |  |  |  |  | AVLT-D | -0.288 | 0.005** |
| Frontal_Sup_R |  |  |  | MMSE | -0.46 | 0.003** |  |  |  |
| Frontal_Sup_Orb_L | MMSE | 0.3 | 0.029* |  |  |  |  |  |  |
| Frontal_Sup_Orb_R | AVLT-D | 0.321 | 0.019* |  |  |  | AVLT-D | 0.209 | 0.044* |
|  | MMSE | 0.385 | 0.004** |  |  |  | MMSE | 0.29 | 0.005** |
|  |  |  |  |  |  |  | MoCA | 0.238 | 0.03* |
| Frontal_Inf_Oper_L | MMSE | -0.34 | 0.013* |  |  |  | MMSE | -0.235 | 0.023* |
| Frontal_Inf_Tri_L |  |  |  | MMSE | -0.321 | 0.043* | MMSE | -0.22 | 0.034* |
|  |  |  |  |  |  |  | MoCA | -0.24 | 0.029* |
| Frontal_Inf_Orb_L | AVLT-D | 0.356 | 0.009** | MMSE | -0.454 | 0.003** |  |  |  |
|  | AVLT-R | 0.344 | 0.012* |  |  |  |  |  |  |
|  | MoCA | 0.313 | 0.034* |  |  |  |  |  |  |
| Frontal_Inf_Orb_R | AVLT-D | 0.284 | 0.039* |  |  |  | AVLT-R | 0.228 | 0.028* |
|  | AVLT-R | 0.311 | 0.024* |  |  |  |  |  |  |
| Rolandic_Oper_L |  |  |  | MoCA | -0.327 | 0.048* |  |  |  |
| Supp_Motor_Area_L |  |  |  |  |  |  | MMSE | 0.213 | 0.04* |
| Olfactory_L | MoCA | 0.411 | 0.005** |  |  |  |  |  |  |
| Frontal_Mid_Orb_R |  |  |  |  |  |  | MoCA | 0.243 | 0.027* |
| Rectus_R | MMSE | 0.288 | 0.037* | MoCA | 0.336 | 0.042* | MoCA | 0.29 | 0.008** |
| Cingulum_Ant_L |  |  |  |  |  |  | AVLT-I | -0.251 | 0.016* |
| Cingulum_Mid_R | AVLT-D | -0.277 | 0.044* |  |  |  |  |  |  |
|  | AVLT-R | -0.28 | 0.042* |  |  |  |  |  |  |
| Calcarine_L |  |  |  | AVLT-D | -0.359 | 0.023* |  |  |  |
|  |  |  |  | AVLT-R | -0.334 | 0.035* |  |  |  |
| Calcarine_R |  |  |  | AVLT-D | -0.448 | 0.004** |  |  |  |
| Cuneus_L |  |  |  |  |  |  | AVLT-D | -0.216 | 0.037* |
| Cuneus_R |  |  |  | AVLT-D | -0.418 | 0.007** |  |  |  |
| Lingual_L |  |  |  | AVLT-D | -0.368 | 0.019* |  |  |  |
| Lingual_R | AVLT-R | 0.28 | 0.042* | AVLT-D | -0.424 | 0.006** |  |  |  |
| Occipital_Sup_R |  |  |  | AVLT-D | -0.343 | 0.03* |  |  |  |
| Fusiform_L |  |  |  | AVLT-R | -0.337 | 0.034* |  |  |  |
|  |  |  |  | MMSE | -0.339 | 0.032* |  |  |  |
| Fusiform_R |  |  |  |  |  |  | AVLT-D | -0.267 | 0.01** |
| Postcentral_L | AVLT-D | -0.389 | 0.004** |  |  |  | AVLT-D | -0.287 | 0.005** |
| Parietal_Sup_R |  |  |  | AVLT-D | -0.378 | 0.016* | MMSE | -0.204 | 0.049* |
|  |  |  |  | MMSE | -0.362 | 0.022* |  |  |  |
| Parietal_Inf_R |  |  |  | AVLT-D | -0.378 | 0.016* |  |  |  |
|  |  |  |  | MMSE | -0.324 | 0.041* |  |  |  |
| Angular_R |  |  |  |  |  |  | MMSE | -0.212 | 0.041* |
|  |  |  |  |  |  |  | MoCA | -0.223 | 0.043* |
| Precuneus_L | AVLT-R | 0.279 | 0.043* |  |  |  | MMSE | -0.235 | 0.023* |
| Precuneus_R |  |  |  | AVLT-D | -0.401 | 0.01** | AVLT-D | -0.219 | 0.035* |
|  |  |  |  | MMSE | -0.379 | 0.016* |  |  |  |
| Caudate_L | AVLT-I | 0.343 | 0.012* | AVLT-D | -0.328 | 0.039* |  |  |  |
|  | AVLT-D | 0.316 | 0.021* |  |  |  |  |  |  |
| Caudate_R | AVLT-I | 0.372 | 0.006** | AVLT-D | -0.331 | 0.037* |  |  |  |
|  | AVLT-D | 0.323 | 0.018* |  |  |  |  |  |  |
|  | AVLT-R | 0.325 | 0.017* |  |  |  |  |  |  |
| Putamen_R | AVLT-I | 0.331 | 0.015* | MMSE | 0.332 | 0.036* | MMSE | 0.226 | 0.03* |
|  |  |  |  | MoCA | 0.34 | 0.04* |  |  |  |
| Pallidum_L |  |  |  | AVLT-D | -0.383 | 0.015* |  |  |  |
|  |  |  |  | AVLT-R | -0.356 | 0.024* |  |  |  |
| Pallidum_R | AVLT-I | 0.339 | 0.013* |  |  |  | MMSE | 0.221 | 0.033* |
|  | MMSE | 0.273 | 0.048* |  |  |  |  |  |  |
| Thalamus_L | MoCA | -0.293 | 0.048* |  |  |  |  |  |  |
| Thalamus_R |  |  |  |  |  |  | AVLT-D | -0.226 | 0.029* |
| Temporal_Sup_L |  |  |  | AVLT-R | -0.389 | 0.013* |  |  |  |
|  |  |  |  | MoCA | -0.395 | 0.015* |  |  |  |
| Temporal_Mid_L |  |  |  | MMSE | -0.421 | 0.007** |  |  |  |
| Temporal_Mid_R |  |  |  | AVLT-D | -0.337 | 0.034* | AVLT-D | -0.216 | 0.038* |
| Temporal_Pole_Mid_R | MoCA | 0.385 | 0.008** |  |  |  | MoCA | 0.277 | 0.011* |

The results were based on the linear regression model, with age and education as covariates. P*<0.05 P**<0.01

## Supplementary Table 2: Linear regression model results of dynamic functional connectivity with the threshold of r > 0.25.

| Brain regions | NC | | | SCD | | | All participants | | |
| --- | --- | --- | --- | --- | --- | --- | --- | --- | --- |
|  | Neuropsychology test | Standardized-β | p-values | Neuropsychology test | Standardized-β | p-values | Neuropsychology test | Standardized-β | p-values |
| Frontal_Sup_R |  |  |  |  |  |  | AVLT-R | 0.21 | 0.043* |
| Frontal_Sup_Orb_L | MMSE | 0.275 | 0.047* | MoCA | 0.336 | 0.042* |  |  |  |
| Frontal_Sup_Orb_R | AVLT-D | 0.326 | 0.017* |  |  |  | AVLT-D | 0.242 | 0.019* |
|  | MMSE | 0.299 | 0.03* |  |  |  | MMSE | 0.268 | 0.01** |
| Frontal_Mid_Orb_L | AVLT-D | 0.301 | 0.029* |  |  |  | AVLT-D | 0.278 | 0.007** |
| Frontal_Mid_Orb_R | AVLT-D | 0.375 | 0.006** |  |  |  | AVLT-D | 0.337 | 0.001** |
| Frontal_Inf_Oper_L | MMSE | -0.281 | 0.042* |  |  |  | MMSE | -0.269 | 0.009** |
|  |  |  |  |  |  |  | MoCA | -0.242 | 0.027* |
| Frontal_Inf_Tri_L |  |  |  |  |  |  | MoCA | -0.247 | 0.025* |
| Frontal_Inf_Orb_L |  |  |  | MMSE | -0.484 | 0.002** |  |  |  |
| Rolandic_Oper_L |  |  |  | MoCA | -0.448 | 0.005** |  |  |  |
| Supp_Motor_Area_L | AVLT-I | -0.312 | 0.023* | MMSE | 0.37 | 0.019* | AVLT-I | -0.222 | 0.034* |
|  |  |  |  |  |  |  | MMSE | 0.238 | 0.022* |
| Supp_Motor_Area_R |  |  |  | MMSE | 0.315 | 0.048* | MMSE | 0.244 | 0.018* |
| Olfactory_L | MoCA | 0.37 | 0.011* | MMSE | 0.389 | 0.013* | MMSE | 0.315 | 0.002** |
|  |  |  |  |  |  |  | MoCA | 0.216 | 0.05* |
| Frontal_Sup_Medial_L | AVLT-I | 0.299 | 0.03* |  |  |  |  |  |  |
| Frontal_Sup_Medial_R | AVLT-D | 0.271 | 0.05* |  |  |  |  |  |  |
| Frontal_Mid_Orb_R | MMSE | 0.303 | 0.027* |  |  |  | MMSE | 0.211 | 0.043* |
|  | MoCA | 0.339 | 0.021* |  |  |  | MoCA | 0.253 | 0.021* |
| Insula_L | AVLT-R | 0.344 | 0.012* |  |  |  |  |  |  |
| Cingulum_Mid_L | MoCA | -0.42 | 0.004** |  |  |  | MoCA | -0.257 | 0.019* |
| Cingulum_Mid_R | MoCA | -0.299 | 0.044* |  |  |  |  |  |  |
| Amygdala_L |  |  |  | MMSE | 0.385 | 0.014* | MMSE | 0.208 | 0.045* |
| Calcarine_L |  |  |  | MoCA | -0.456 | 0.005** |  |  |  |
| Cuneus_L |  |  |  | MoCA | -0.359 | 0.029* |  |  |  |
| Cuneus_R |  |  |  | AVLT-D | -0.382 | 0.015* | MMSE | -0.241 | 0.02* |
|  |  |  |  | AVLT-R | -0.36 | 0.023* |  |  |  |
|  |  |  |  | MMSE | -0.391 | 0.013* |  |  |  |
|  |  |  |  | MoCA | -0.395 | 0.015* |  |  |  |
| Lingual_R | AVLT-D | -0.32 | 0.019* |  |  |  | AVLT-D | -0.232 | 0.025* |
| Occipital_Sup_R |  |  |  |  |  |  | AVLT-R | -0.215 | 0.039* |
| Occipital_Mid_R |  |  |  | MMSE | -0.315 | 0.048* | AVLT-R | -0.232 | 0.026* |
|  |  |  |  |  |  |  | MMSE | -0.206 | 0.048* |
| Occipital_Inf_L |  |  |  |  |  |  | MoCA | 0.225 | 0.041* |
| Fusiform_L |  |  |  | MoCA | -0.426 | 0.009** |  |  |  |
| Postcentral_L | AVLT-R | -0.272 | 0.049* |  |  |  |  |  |  |
| Parietal_Sup_L | MoCA | -0.371 | 0.011* |  |  |  |  |  |  |
| Parietal_Inf_L | MMSE | -0.29 | 0.035* |  |  |  |  |  |  |
|  | MoCA | -0.445 | 0.002** |  |  |  |  |  |  |
| Parietal_Inf_R | MoCA | -0.303 | 0.041* |  |  |  |  |  |  |
| SupraMarginal_L |  |  |  | MMSE | -0.48 | 0.002** |  |  |  |
| Angular_L |  |  |  | AVLT-D | 0.329 | 0.038* | AVLT-D | 0.259 | 0.012* |
|  |  |  |  | AVLT-R | 0.325 | 0.041* |  |  |  |
| Angular_R |  |  |  | MoCA | -0.509 | 0.001** | MoCA | -0.246 | 0.025* |
| Paracentral_Lobule_L | MoCA | -0.333 | 0.024* |  |  |  |  |  |  |
| Paracentral_Lobule_R | AVLT-I | 0.378 | 0.005** | AVLT-D | 0.349 | 0.028* | AVLT-I | 0.217 | 0.038* |
|  |  |  |  | MMSE | 0.416 | 0.008** | AVLT-D | 0.305 | 0.003** |
|  |  |  |  |  |  |  | MMSE | 0.22 | 0.034* |
| Caudate_L | AVLT-R | 0.276 | 0.045* |  |  |  |  |  |  |
| Thalamus_L | AVLT-D | -0.325 | 0.018* |  |  |  |  |  |  |
|  | MoCA | -0.389 | 0.008** |  |  |  |  |  |  |
| Thalamus_R | MoCA | -0.454 | 0.002** | MMSE | 0.329 | 0.038* |  |  |  |
| Heschl_L |  |  |  | MMSE | -0.405 | 0.01** | MMSE | -0.214 | 0.04* |
|  |  |  |  | MoCA | -0.438 | 0.007** |  |  |  |
| Temporal_Sup_L |  |  |  | AVLT-R | -0.337 | 0.033* | AVLT-R | -0.214 | 0.039* |
|  |  |  |  | MoCA | -0.433 | 0.007** |  |  |  |
| Temporal_Pole_Sup_R |  |  |  | AVLT-I | -0.359 | 0.025* |  |  |  |
| Temporal_Mid_L |  |  |  | MMSE | -0.508 | 0.001** | MMSE | -0.229 | 0.027* |
|  |  |  |  | MoCA | -0.44 | 0.006** |  |  |  |
| Temporal_Mid_R |  |  |  | AVLT-D | -0.318 | 0.046* |  |  |  |
|  |  |  |  | MMSE | -0.424 | 0.006** |  |  |  |
|  |  |  |  | MoCA | -0.371 | 0.024* |  |  |  |
| Temporal_Pole_Mid_R | MoCA | 0.313 | 0.034* | AVLT-I | 0.36 | 0.024* | AVLT-I | 0.226 | 0.03* |

The results were based on the linear regression model, with age and education as covariates. P*<0.05 P**<0.01

## Supplementary Table 3: Linear regression model results of dynamic functional connectivity with the threshold of r > 0.2.

| Brain regions | NC | | | SCD | | |
| --- | --- | --- | --- | --- | --- | --- |
|  | Neuropsychology test | Standardized-β | p-values | Neuropsychology test | Standardized-β | p-values |
| Precentral_R | MoCA | -0.301 | 0.042* |  |  |  |
| Frontal_Sup_Orb_R | AVLT-D | 0.335 | 0.014* |  |  |  |
|  | MMSE | 0.312 | 0.023* |  |  |  |
| Frontal_Mid_Orb_L | AVLT-D | 0.287 | 0.037* |  |  |  |
| Frontal_Mid_Orb_R | AVLT-I | 0.284 | 0.039* | MoCA | 0.35 | 0.034* |
|  | AVLT-D | 0.38 | 0.005** |  |  |  |
| Frontal_Inf_Oper_L | MMSE | -0.312 | 0.023* |  |  |  |
| Frontal_Inf_Tri_R | MoCA | -0.312 | 0.035* |  |  |  |
| Frontal_Inf_Orb_L |  |  |  | MMSE | -0.476 | 0.002** |
| Rolandic_Oper_L |  |  |  | MoCA | -0.415 | 0.011* |
| Supp_Motor_Area_L | AVLT-I | -0.312 | 0.023* | MMSE | 0.331 | 0.037* |
| Olfactory_L | MoCA | 0.38 | 0.009** | MMSE | 0.365 | 0.02* |
| Frontal_Sup_Medial_L | AVLT-I | 0.291 | 0.034* |  |  |  |
| Frontal_Sup_Medial_R | AVLT-I | 0.303 | 0.027* |  |  |  |
|  | AVLT-D | 0.34 | 0.013* |  |  |  |
| Frontal_Mid_Orb_R | MMSE | 0.289 | 0.036* |  |  |  |
|  | MoCA | 0.335 | 0.023* |  |  |  |
| Insula_L | AVLT-R | 0.358 | 0.008** |  |  |  |
| Insula_R | AVLT-R | 0.337 | 0.014* |  |  |  |
| Cingulum_Mid_L | MoCA | -0.39 | 0.007** |  |  |  |
| Cingulum_Mid_R | MoCA | -0.298 | 0.044* |  |  |  |
| Amygdala_L |  |  |  | MMSE | 0.357 | 0.024* |
| Calcarine_L |  |  |  | MoCA | -0.447 | 0.006** |
| Cuneus_R |  |  |  | AVLT-D | -0.358 | 0.023* |
|  |  |  |  | AVLT-R | -0.344 | 0.03* |
|  |  |  |  | MMSE | -0.346 | 0.029* |
|  |  |  |  | MoCA | -0.354 | 0.032* |
| Lingual_R | AVLT-D | -0.327 | 0.017* |  |  |  |
| Occipital_Sup_R | AVLT-R | -0.273 | 0.048* |  |  |  |
| Occipital_Mid_L |  |  |  | MMSE | -0.336 | 0.034* |
| Occipital_Mid_R |  |  |  | MMSE | -0.319 | 0.045* |
| Fusiform_L |  |  |  | MoCA | -0.412 | 0.011* |
| Postcentral_L | AVLT-R | -0.271 | 0.05* | MMSE | 0.322 | 0.043* |
| Parietal_Sup_L | MoCA | -0.323 | 0.029* |  |  |  |
| Parietal_Inf_L | MMSE | -0.293 | 0.033* |  |  |  |
|  | MoCA | -0.441 | 0.002** |  |  |  |
| Parietal_Inf_R | MoCA | -0.304 | 0.04* |  |  |  |
| SupraMarginal_L |  |  |  | MMSE | -0.516 | 0.001** |
| Angular_L |  |  |  | AVLT-D | 0.328 | 0.039* |
| Angular_R |  |  |  | MoCA | -0.458 | 0.004** |
| Paracentral_Lobule_L | MoCA | -0.383 | 0.009** |  |  |  |
| Paracentral_Lobule_R | AVLT-I | 0.357 | 0.009** | AVLT-D | 0.344 | 0.03* |
|  |  |  |  | MMSE | 0.444 | 0.004** |
| Thalamus_L | AVLT-D | -0.308 | 0.025* |  |  |  |
|  | MoCA | -0.354 | 0.016* |  |  |  |
| Thalamus_R | MoCA | -0.433 | 0.003** | MMSE | 0.32 | 0.044* |
| Heschl_L |  |  |  | MMSE | -0.41 | 0.009** |
|  |  |  |  | MoCA | -0.462 | 0.004** |
| Temporal_Sup_L |  |  |  | MoCA | -0.392 | 0.016* |
| Temporal_Pole_Sup_R |  |  |  | AVLT-I | -0.364 | 0.023* |
| Temporal_Mid_L |  |  |  | AVLT-R | -0.321 | 0.043* |
|  |  |  |  | MMSE | -0.498 | 0.001** |
|  |  |  |  | MoCA | -0.379 | 0.021* |
| Temporal_Mid_R |  |  |  | MMSE | -0.411 | 0.008** |
|  |  |  |  | MoCA | -0.327 | 0.048* |
| Temporal_Pole_Mid_R | MoCA | 0.319 | 0.031* |  |  |  |
| Temporal_Inf_L |  |  |  | MoCA | -0.327 | 0.048* |

The results were based on the linear regression model, with age and education as covariates. P*<0.05 P**<0.01

## Supplementary Table 4: Linear regression model results of static functional connectivity with the sparsity of 0.2.

| Brain regions | NC | | | SCD | | |
| --- | --- | --- | --- | --- | --- | --- |
|  | Neuropsychology test | Standardized-β | p-values | Neuropsychology test | Standardized-β | p-values |
| Precentral_L | MMSE | -0.292 | 0.034* |  |  |  |
| Frontal_Sup_Orb_R | AVLT-I | 0.286 | 0.038* |  |  |  |
|  | AVLT-D | 0.356 | 0.009** |  |  |  |
|  | MMSE | 0.345 | 0.011* |  |  |  |
| Frontal_Inf_Oper_L | MMSE | -0.403 | 0.003** |  |  |  |
| Frontal_Inf_Orb_L | AVLT-D | 0.423 | 0.002** | MMSE | -0.492 | 0.001** |
|  | AVLT-R | 0.302 | 0.028* | MoCA | -0.332 | 0.044* |
| Frontal_Inf_Orb_R | AVLT-D | 0.353 | 0.01** |  |  |  |
|  | AVLT-R | 0.305 | 0.027* |  |  |  |
| Rolandic_Oper_L |  |  |  | MoCA | -0.413 | 0.011* |
| Olfactory_L | MoCA | 0.393 | 0.007** |  |  |  |
| Olfactory_R | MoCA | 0.294 | 0.047* |  |  |  |
| Rectus_R | MMSE | 0.28 | 0.042* |  |  |  |
| Cingulum_Mid_L | AVLT-D | -0.275 | 0.046* |  |  |  |
|  | AVLT-R | -0.279 | 0.043* |  |  |  |
|  | MoCA | -0.305 | 0.039* |  |  |  |
| Cingulum_Mid_R | AVLT-R | -0.396 | 0.003** |  |  |  |
| Calcarine_R |  |  |  | AVLT-D | -0.369 | 0.019* |
| Cuneus_R |  |  |  | AVLT-D | -0.371 | 0.018* |
| Lingual_L |  |  |  | AVLT-D | -0.352 | 0.026* |
| Lingual_R | AVLT-R | 0.311 | 0.024* | AVLT-D | -0.395 | 0.012* |
| Occipital_Inf_R |  |  |  | MMSE | 0.344 | 0.03* |
| Fusiform_L |  |  |  | MMSE | -0.379 | 0.016* |
| Postcentral_L | AVLT-D | -0.38 | 0.005** |  |  |  |
|  | MMSE | -0.272 | 0.049* |  |  |  |
| Parietal_Sup_L | MoCA | -0.34 | 0.021* |  |  |  |
| Parietal_Inf_R | MoCA | -0.35 | 0.017* |  |  |  |
| Angular_R | AVLT-R | -0.287 | 0.037* |  |  |  |
| Paracentral_Lobule_L | MoCA | -0.304 | 0.04* |  |  |  |
| Paracentral_Lobule_R | AVLT-I | 0.28 | 0.042* |  |  |  |
| Caudate_R | AVLT-I | 0.365 | 0.007** |  |  |  |
|  | AVLT-D | 0.282 | 0.041* |  |  |  |
|  | AVLT-R | 0.292 | 0.034* |  |  |  |
| Putamen_L |  |  |  | MMSE | 0.369 | 0.019* |
| Putamen_R |  |  |  | MMSE | 0.341 | 0.031* |
| Pallidum_R | AVLT-I | 0.359 | 0.008** |  |  |  |
| Thalamus_R | AVLT-D | -0.275 | 0.046* |  |  |  |
| Temporal_Mid_L |  |  |  | MMSE | -0.38 | 0.016* |
| Temporal_Pole_Mid_R | MoCA | 0.339 | 0.021* | AVLT-D | 0.316 | 0.047* |
| Temporal_Inf_R | AVLT-R | -0.333 | 0.015* | AVLT-D | 0.324 | 0.042* |

The results were based on the linear regression model, with age and education as covariates. P*<0.05 P**<0.01

## Supplementary Table 5: Linear regression model results of static functional connectivity with the sparsity of 0.25.

| Brain regions | NC | | | SCD | | |
| --- | --- | --- | --- | --- | --- | --- |
|  | Neuropsychology test | Standardized-β | p-values | Neuropsychology test | Standardized-β | p-values |
| Precentral_L | MMSE | -0.275 | 0.046* |  |  |  |
| Frontal_Sup_R |  |  |  | MMSE | -0.352 | 0.026* |
| Frontal_Sup_Orb_R | AVLT-I | 0.303 | 0.028* |  |  |  |
|  | AVLT-D | 0.379 | 0.005** |  |  |  |
|  | MMSE | 0.346 | 0.011* |  |  |  |
| Frontal_Inf_Oper_L | MMSE | -0.381 | 0.005** |  |  |  |
| Frontal_Inf_Tri_R |  |  |  | AVLT-R | 0.318 | 0.045* |
| Frontal_Inf_Orb_L | AVLT-D | 0.318 | 0.02* | MMSE | -0.409 | 0.009** |
| Frontal_Inf_Orb_R | AVLT-D | 0.341 | 0.013* |  |  |  |
|  | AVLT-R | 0.291 | 0.034* |  |  |  |
| Rolandic_Oper_L |  |  |  | MoCA | -0.345 | 0.037* |
| Olfactory_L | MoCA | 0.354 | 0.016* |  |  |  |
| Olfactory_R | MoCA | 0.292 | 0.049* |  |  |  |
| Cingulum_Mid_L | MoCA | -0.302 | 0.042* |  |  |  |
| Cingulum_Mid_R | AVLT-D | -0.291 | 0.035* |  |  |  |
|  | AVLT-R | -0.354 | 0.009** |  |  |  |
|  | MoCA | -0.358 | 0.015* |  |  |  |
| Calcarine_L |  |  |  | AVLT-D | -0.334 | 0.035* |
|  |  |  |  | AVLT-R | -0.327 | 0.04* |
| Calcarine_R |  |  |  | AVLT-D | -0.367 | 0.02* |
| Lingual_L |  |  |  | AVLT-D | -0.336 | 0.034* |
| Lingual_R |  |  |  | AVLT-D | -0.372 | 0.018* |
| Postcentral_L | AVLT-I | -0.277 | 0.045* |  |  |  |
|  | AVLT-D | -0.382 | 0.005** |  |  |  |
| Parietal_Sup_L | MoCA | -0.335 | 0.023* |  |  |  |
| Parietal_Inf_R | MoCA | -0.322 | 0.029* |  |  |  |
| Angular_R | AVLT-R | -0.357 | 0.009** |  |  |  |
| Precuneus_L | MMSE | -0.272 | 0.049* |  |  |  |
| Paracentral_Lobule_L | MoCA | -0.316 | 0.033* |  |  |  |
| Paracentral_Lobule_R | AVLT-I | 0.308 | 0.025* |  |  |  |
| Caudate_L | AVLT-I | 0.375 | 0.006** |  |  |  |
|  | AVLT-D | 0.369 | 0.007** |  |  |  |
|  | AVLT-R | 0.288 | 0.037* |  |  |  |
| Caudate_R | AVLT-I | 0.324 | 0.018* |  |  |  |
|  | AVLT-R | 0.273 | 0.048* |  |  |  |
| Putamen_L |  |  |  | MMSE | 0.326 | 0.04* |
| Putamen_R | AVLT-I | 0.319 | 0.02* | MMSE | 0.376 | 0.017* |
|  |  |  |  | MoCA | 0.354 | 0.031* |
| Pallidum_R | AVLT-I | 0.276 | 0.046* |  |  |  |
| Thalamus_L | MoCA | -0.332 | 0.024* |  |  |  |
| Thalamus_R | AVLT-D | -0.303 | 0.027* |  |  |  |
| Temporal_Sup_L |  |  |  | MoCA | -0.449 | 0.005** |
| Temporal_Pole_Sup_L | AVLT-R | 0.319 | 0.02* |  |  |  |
| Temporal_Mid_L |  |  |  | MMSE | -0.37 | 0.019* |
|  |  |  |  | MoCA | -0.325 | 0.05* |
| Temporal_Pole_Mid_R | MoCA | 0.354 | 0.016* | AVLT-D | 0.332 | 0.036* |
| Temporal_Inf_R | AVLT-R | -0.354 | 0.009** | AVLT-D | 0.358 | 0.023* |

The results were based on the linear regression model, with age and education as covariates. P^*^<0.05 P^**^<0.01
